# Microplastics in focus: a silent disruptor of liver health- a systematic review

**DOI:** 10.3389/fphar.2025.1721644

**Published:** 2025-12-01

**Authors:** Zahra Beyzaei, Bita Geramizadeh, Zahra Bagheri, Sara Karimzadeh, Ralf Weiskirchen

**Affiliations:** 1 Transplant Research Center, Shiraz University of Medical Sciences, Shiraz, Iran; 2 Department of Pathology, Medical School of Shiraz University, Shiraz University of Medical Sciences, Shiraz, Iran; 3 Harvard Medical School and Massachusetts General Hospital, Boston, MA, United States; 4 Shiraz Medical School Library, Shiraz University of Medical Sciences, Shiraz, Iran; 5 Institute of Molecular Pathobiochemistry, Experimental Gene Therapy and Clinical Chemistry (IFMPEGKC) RWTH University Hospital Aachen, Aachen, Germany

**Keywords:** microplastics, polystyrene, hepatotoxicity, liver organoids, human liver health

## Abstract

Micro- and nanoplastics (MNPs) are widespread environmental contaminants, yet their impact on human liver health is not fully understood. We conducted a systematic review of 25 experimental, observational, and organoid-based studies published between 2022 and 2025 that investigated the hepatotoxic effects of polystyrene micro- and nanoplastics (PS-MPs/NPs). Following PRISMA guidelines, we screened 770 records from PubMed, EMBASE, Scopus, and Web of Science. After removing duplicates, conducting dual-stage screening, and assessing quality using the Newcastle–Ottawa Scale, 25 studies met our predefined inclusion criteria. Seventeen studies using human liver-derived cell lines consistently reported oxidative stress, inflammation, apoptosis, mitochondrial dysfunction, and disturbances in lipid-metabolism in a size- and dose-dependent manner, with nanoplastics showing the highest toxicity. Six investigations using pluripotent-stem-cell-derived liver organoids confirmed and expanded upon these findings, demonstrating that both pristine and aged PS-MPs (1–10 µm) disrupt sulfur amino acid and iron homeostasis (e.g., increased serum cysteine, decreased hepatic cysteine, and disturbed homocysteine metabolism), impair mitochondrial bioenergetics, and lead to significant lipid accumulation after exposures lasting up to 500 h. Limited human evidence indicated transplacental transfer of PS-MP associated with elevated fetal liver enzymes (alkaline phosphatase, aspartate aminotransferase, and γ-glutamyl transferase) in 1,057 pregnancies, and higher microplastic levels were found in cirrhotic livers compared to non-diseased livers, underscoring potential clinical implications. Current findings suggest that exposure to PS-MP/NP disrupts hepatic redox balance, metabolic function, and structural integrity across *in vitro*, organoid, and human models. However, variability in particle characterization, exposure methods, and outcome measures, along with limited epidemiological data, hinder definitive risk assessment. Future research should prioritize standardized methodologies, longitudinal human studies, and advanced mechanistic models to establish exposure thresholds and develop strategies to mitigate microplastic-induced hepatotoxicity.

**Systematic Review Registration:** https://www.crd.york.ac.uk/PROSPERO/view/CRD420251159265.

## 1 Introduction

Microplastics (MPs) are defined as plastic fragments ≤5 mm in size, while nanoplastics (NPs) are generally considered to be ≤ 1 μm (Otorkpa and Otorkpa, 2024). Despite global initiatives to reduce plastic production and enhance recycling, projections estimate that between 2016 and 2040, approximately 250 million metric tons (Mt) of plastic waste will enter aquatic systems and 460 million Mt will accumulate in terrestrial environments (Liu et al., 2024; Haleem et al., 2024; Dzierżyński et al., 2024). Humans are continuously and unavoidably exposed to micro- and nanoplastics (MNPs), raising serious concerns about their potential health risks (Feng et al., 2023; Das, 2023; Zolotova et al., 2022; Xu et al., 2022). However, the direct impact of MNPs on human health remains unclear, largely due to challenges in human tissue sampling, the lack of robust epidemiological studies, and limitations in in situ detection methodologies. Evidence to date suggests that MNPs exert both particulate toxicity and chemical toxicity, highlighting their complex biological effects (Sorci and Loiseau, 2022). MNPs can enter the human body via inhalation, ingestion, and trophic transfer, subsequently accumulating in organs and tissues, where they may disrupt physiological function and pose significant health risks (Garcia et al., 2024; Dubey et al., 2022). Notably, particles <150 μm are capable of translocating from the intestinal lumen into the lymphatic and circulatory systems, ultimately reaching critical organs, including the liver, kidneys, and brain, where they elicit diverse toxic responses (Li et al., 2022).

The liver, as the central metabolic organ, plays a pivotal role in detoxification and is a major site for the accumulation of environmental pollutants. Pollutant enrichment can interfere with liver development and function, with different hepatocyte populations exhibiting variable susceptibility to toxicants. However, there is a lack of well-established experimental models that reliably capture the mechanisms through which environmental pollutants, including MNPs, disrupt human liver biology. Emerging technologies, such as stem cell-derived hepatocytes and liver organoids, represent promising platforms to investigate these mechanisms in greater depth. To date, though, research elucidating the specific pathways by which MPs and NPs compromise hepatic barriers and functionality remains scarce (Zhou et al., 2025; Zhang Y. et al., 2025; Ruiz-Ramos et al., 2025; An et al., 2025; Zhang et al., 2024; Sangwan et al., 2024).

In this systematic review, we comprehensively evaluate studies investigating the effects of MNPs on liver development and function, with a focus on the toxicity of these particles in different hepatic cell types. Our synthesis highlights current knowledge gaps and provides a framework for future research into the mechanisms underlying MNP-induced hepatotoxicity. Polystyrene was chosen for this review because of its common presence in the environment, large production volume, and frequent use in human and experimental studies. This makes it a suitable model for evaluating the toxicity of MPs on the liver.

## 2 Methodology

This systematic review was conducted in accordance with the Cochrane Handbook for Systematic Reviews of Interventions and reported following the Preferred Reporting Items for Systematic Reviews and Meta-Analyses (PRISMA) guidelines (Liberati et al., 2009). The review protocol was prospectively registered in PROSPERO (registration no. CRD420251159265). A completed PRISMA checklist is provided in the Supplementary Material (Supplementary Table S1).

### 2.1 Data sources and search strategy

Two authors (ZBe and SK) independently conducted a systematic literature search across MEDLINE/PubMed, EMBASE, Scopus, and Web of Science Core Collection, on 1 September 2025, with no restrictions on language or publication date (from database inception to that date). To minimize publication bias, the reference lists of all included studies and relevant systematic reviews were also manually screened for additional eligible articles.

The search strategy was developed using both controlled vocabulary (MeSH terms) and free-text keywords, in consultation with an experienced information specialist (SK), who has expertise in systematic review methodology and database searching. Keywords were identified through examination of relevant references and validated Medical Subject Headings (http://www.nlm.nih.gov/mesh/). The primary search terms included: “Plastic Microparticle” OR Mesoplastic OR “Plastic Nanoparticle” OR Microplastic* OR Micro-plastic* OR “Plastic Debris” AND (“Liver Disease*” OR “Liver Dysfunction*” OR “Liver Health” OR “Hepatic Disease*” OR Hepatotoxicity) (Table 1).

**TABLE 1:** Search strategy conducted across all databases on 1 September 2025.

Databases	Controlled vocabulary (MeSH terms) and free-text keywords	No. paper
PubMed	(“plastic microparticle*” [Title/Abstract] OR “mesoplastic*” [Title/Abstract] OR “plastic nanoparticle*” [Title/Abstract] OR “microplastic*” [Title/Abstract] OR “micro plastic*” [Title/Abstract] OR “plastic debris” [Title/Abstract] OR “Microplastics” [MeSH Terms]) AND (“liver disease*” [Title/Abstract] OR “liver dysfunction*” [Title/Abstract] OR “liver health” [Title/Abstract] OR “hepatic disease*” [Title/Abstract] OR “Hepatotoxicity” [Title/Abstract] OR (“Liver” [MeSH Terms] OR “Liver Diseases” [MeSH Terms]))	326
Scopus	(TITLE-ABS-KEY (“plastic microparticle*” OR mesoplastic* OR “plastic nanoparticle*” OR microplastic* OR micro-plastic* OR “plastic debris”) AND TITLE-ABS-KEY (“liver disease*” OR “liver dysfunction*” OR “liver health” OR “hepatic disease*” OR hepatotoxicity))	170
WOS	“Plastic Microparticle*” OR Mesoplastic* OR “Plastic Nanoparticle*” OR Microplastic* OR Micro-plastic* OR “plastic debris” (Topic) and “Liver Disease*” OR “Liver Dysfunction*” OR “liver health” OR “hepatic disease*” OR Hepatotoxicity (Topic)	152
Embase	(“plastic microparticle*”:ti,ab,kw OR mesoplastic*:ti,ab,kw OR “plastic nanoparticle*”:ti,ab,kw OR microplastic*:ti,ab,kw OR “micro plastic*”:ti,ab,kw OR “plastic debris”:ti,ab,kw) AND (“liver disease*”:ti,ab,kw OR “liver dysfunction*”:ti,ab,kw OR “liver health”:ti,ab,kw OR “hepatic disease*”:ti,ab,kw OR hepatotoxicity:ti,ab,kw)	122

### 2.2 Study selection and criteria

Initially, studies of any relevant design and in any language assessing the impact of MPs and NPs on the human liver were considered.

The inclusion criteria were as follows:

1. Studies explicitly designed to evaluate the association between MPs or NPs and liver-related outcomes.
2. Studies reporting the size and type of MPs and NPs.
3. Studies providing comparative data for cases and controls.
4. Only polystyrene (PS) particle data were extracted. If any article reported single and combined exposure only single data was extracted.
5. Outcomes related to liver function, enzyme activity, cellular toxicity, hepatotoxicity, inflammation or mortality.

The exclusion criteria included:

Case reports, editorials, reviews, letters, conference abstracts, animal studies, and studies not involving liver cells.

Two authors (Z.Be. and B.G.) independently assessed potential eligibility in two sequential stages: first, screening titles and abstracts, followed by full-text evaluation based on the above criteria. Full texts of potentially eligible studies were thoroughly reviewed by both reviewers, and studies meeting all inclusion criteria were selected for detailed data extraction and quality assessment.

Inter-rater reliability was evaluated using Cohen’s kappa statistic with MedCalc software version 19.1. Any discrepancies at any stage were resolved through discussion, with reference to the original articles as needed to reach consensus.

### 2.3 Methodological quality assessment

The methodological quality of the included studies was independently evaluated by two reviewers (Z.Be. and Z.Ba.). The Newcastle-Ottawa Quality Assessment Scale (NOS) for Case-Control Studies was used as the assessment tool. This tool evaluates multiple domains of internal validity, including selection, comparability, and exposure assessment, through a series of 14 criteria. Each criterion was rated as “Yes” if adequately met or “No” if not. Based on the total score, studies were categorized as Good (>8 “Yes” responses), Fair (7–8 “Yes”), or Poor (<6 “Yes”). Overall agreement between the reviewers was high, with 87% concordance (Table 2).

**TABLE 2:** Methodological quality assessment of included studies. New Castle-Ottawa Scale evaluation of the observational studies included in the systematic review (n = 17), stratified by year of publication.

Study	Type of study	Selection	Comparability	Exposure	Sum	Bias item(s)
Maximum score		****	**	****	10	
Zhang et al. (2025b)	*In vitro* studies	****	**	****	10	Assessment of outcome
Xu et al. (2025)	*In vitro* studies	***	*	**	6	Ascertainment of exposure, Assessment of outcome
Shi et al. (2025)	*In vitro* studies	****	**	****	10	
Li et al. (2025a)	*In vitro* studies	***	**	****	9	Selection
Ahn et al. (2025)	*In vitro* studies	****	*	***	8	Ascertainment of exposure, Assessment of outcome
He et al. (2024)	*In vitro* studies	****	**	****	10	
Guraka et al. (2024)	*In vitro* studies	****	*	***	8	
Guo et al. (2024)	*In vitro* studies	***	**	*	6	Ascertainment of exposure, Assessment of outcome
Fan et al. (2024)	*In vitro* studies	***	**	*	6	Ascertainment of exposure, Assessment of outcome
Chen et al. (2024a)	*In vitro* studies	***	*	***	6	Ascertainment of exposure
Boran et al. (2024)	*In vitro* studies	****	**	***	9	Ascertainment of exposure
Li et al. (2023a)	*In vitro* studies	***	**	***	8	Selection control, Ascertainment of exposure
Li et al. (2023b)	*In vitro* studies	****	**	***	9	
Shen et al. (2022)	*In vitro* studies	***	**	**	7	Selection control
Menéndez-Pedriza et al. (2022)	*In vitro* studies	****	*	***	8	Ascertainment of exposure, Assessment of outcome
Lin et al. (2022)	*In vitro* studies	****	**	****	10	
Banerjee et al. (2022)	*In vitro* studies	****	**	***	9	
Cheng et al. (2025)	*In vitro* studies	***	**	****	9	Selection
Liang et al. (2024)	*In vitro* studies	****	**	****	10	
Cheng et al. (2024a)	*In vitro* studies	***	**	****	9	Selection
Cheng et al. (2024b)	*In vitro* studies	***	**	****	9	Selection
Cheng et al. (2023)	*In vitro* studies	***	**	****	9	Selection
Cheng et al. (2022)	*In vitro* studies	***	**	****	9	Selection
Wa et al. (2025)	cohort	****	*	***	9	Follow up
Horvatits et al. (2022)	*In vitro* studies	****	**	****	10	

Since all included studies were observational in design, additional contextual factors, such as study population characteristics and setting, were also considered during the assessment to ensure comprehensive evaluation of methodological rigor.

### 2.4 Data extraction

In accordance with the PRISMA guidelines, data extraction was independently conducted by two authors (Z.Be. and B.G.) using a standardized data extraction form. Any disagreements were initially resolved through consensus. If consensus could not be reached, the original article was re-evaluated in consultation with a third reviewer (R.W.), and discrepancies were resolved through discussion until full agreement was achieved.

For studies investigating multiple MP polymers, only polystyrene-specific data were extracted, as this review specifically focuses on polystyrene in order to provide a consistent and representative synthesis of hepatotoxic effects.

The extracted data included bibliographic information, first author and year of publication, study design, type of MPs, size and shape of MPs, dose/concentration, exposure route, duration, experimental model, liver-related outcome indicators, and main findings. When necessary, reviewers contacted the corresponding authors of selected studies to obtain additional details. All statistical analyses were conducted using Stata IC 15 (College Station, TX, United States).

## 3 Results

### 3.1 Study selection

**FIGURE 1:**
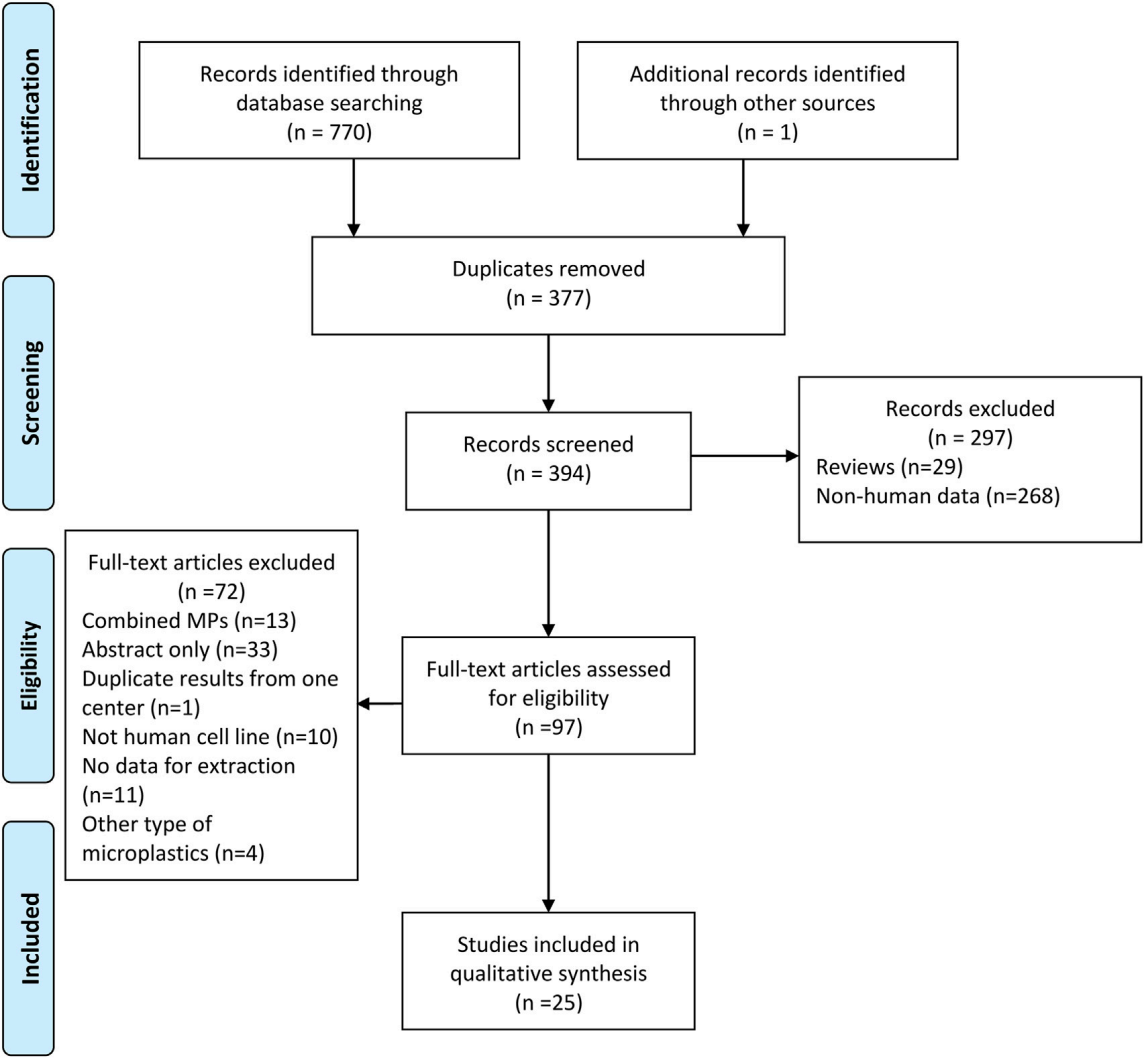
The flow diagram of the study selection for the systematic review.

The study selection process is depicted in the PRISMA flow diagram (Figure 1). The initial database search identified 770 records, of which 377 were duplicates. After removing duplicates, 394 unique articles were identified. Screening of titles and abstracts led to the exclusion of 297 articles based on predefined eligibility criteria, resulting in 97 articles for full-text assessment. Interrater reliability for study selection was high, with a Cohen’s kappa coefficient of 0.85 ± 0.02, indicating strong agreement between the reviewing authors on inclusion and exclusion decisions. Following full-text review and resolution of discrepancies through consensus between two independent reviewers (Z.Be. and B.G.), 25 studies were ultimately included in the systematic review.

### 3.2 General characteristic of study

In total, 25 studies published between 2022 and 2025 were included in this review. All of these studies investigated the hepatotoxic effects of polystyrene MPs (PS-MPs) and NPs (PS-NPs) through various experimental and observational approaches (Zhang H. et al., 2025; Xu et al., 2025; Shi et al., 2025; Li et al., 2025a; Ahn et al., 2025; He et al., 2024; Guraka et al., 2024; Guo et al., 2024; Fan et al., 2024; Chen Z. et al., 2024; Boran et al., 2024; Li et al., 2023a; Li et al., 2023b; Shen et al., 2022; Menéndez-Pedriza et al., 2022; Lin et al., 2022; Banerjee et al., 2022; Cheng et al., 2025; Liang et al., 2024; Cheng et al., 2024a; Cheng et al., 2024b; Cheng et al., 2023; Cheng et al., 2022; Wa et al., 2025; Horvatits et al., 2022). Of these studies, 24 were *in vitro* studies (Zhang H. et al., 2025; Xu et al., 2025; Shi et al., 2025; Li et al., 2025a; Ahn et al., 2025; He et al., 2024; Guraka et al., 2024; Guo et al., 2024; Fan et al., 2024; Chen Z. et al., 2024; Boran et al., 2024; Li et al., 2023a; Li et al., 2023b; Shen et al., 2022; Menéndez-Pedriza et al., 2022; Lin et al., 2022; Banerjee et al., 2022; Cheng et al., 2025; Liang et al., 2024; Cheng et al., 2024a; Cheng et al., 2024b; Cheng et al., 2023; Cheng et al., 2022; Horvatits et al., 2022) and one was a prospective cohort study (Wa et al., 2025). The most frequently used human hepatic cell lines were HepG2 (45%), LO2/L-02 (20%), HepaRG (10%), and THLE-2 (5%), with the remaining studies utilizing other hepatocyte-derived models. Geographically, the majority of these studies were conducted in Asia, with 20 studies originating from countries like China and South Korea (Zhang H. et al., 2025; Xu et al., 2025; Shi et al., 2025; Li et al., 2025a; Ahn et al., 2025; Guo et al., 2024; Fan et al., 2024; Chen Z. et al., 2024; Li et al., 2023a; Li et al., 2023b; Shen et al., 2022; Lin et al., 2022; Cheng et al., 2025; Liang et al., 2024; Cheng et al., 2024a; Cheng et al., 2024b; Cheng et al., 2023; Cheng et al., 2022; Wa et al., 2025). The remaining five studies were from Western countries, including the United States, Germany, Spain, Britain, and Turkey (Guraka et al., 2024; Boran et al., 2024; Menéndez-Pedriza et al., 2022; Banerjee et al., 2022; Horvatits et al., 2022).

Most of the investigations used *in vitro* human liver-derived cell lines such as HepG2, THLE-2, and HepaRG to assess hepatotoxic endpoints (Zhang H. et al., 2025; Xu et al., 2025; Shi et al., 2025; Li et al., 2025a; Ahn et al., 2025; He et al., 2024; Guraka et al., 2024; Guo et al., 2024; Fan et al., 2024; Chen Z. et al., 2024; Boran et al., 2024; Li et al., 2023a; Li et al., 2023b; Shen et al., 2022; Menéndez-Pedriza et al., 2022; Lin et al., 2022; Banerjee et al., 2022). A smaller number of studies utilized human liver organoids derived from pluripotent stem cells or bioprinting techniques, which provided more physiologically relevant models (Cheng et al., 2025; Liang et al., 2024; Cheng et al., 2024a; Cheng et al., 2024b; Cheng et al., 2023; Cheng et al., 2022). Additionally, two human studies were identified: one examining placental exposure and another investigating cirrhotic liver tissue (Wa et al., 2025; Horvatits et al., 2022). Across all studies, polystyrene was consistently selected as the reference polymer, with particle sizes ranging from 20 nm (NPs) to approximately 50 μm (MPs). Three studies specifically evaluated aged PS particles (Cheng et al., 2025; Cheng et al., 2024a; Cheng et al., 2024b). Reported exposure concentrations varied substantially, from environmentally relevant ng/mL levels to supra-environmental mg/mL doses. The majority of *in vitro* studies employed short-term exposures of 24–72 h, while organoid-based investigations extended up to 500 h, allowing for the assessment of longer-term metabolic and mitochondrial effects.

### 3.3 Synthesis of results

#### 3.3.1 Toxicological impacts of microplastics on human liver-derived cell models

A summary of *in vitro* studies published between 2022 and 2025 examining the hepatotoxic effects of PS-MPs and PS-NPs in human liver-derived cell models is presented in Table 3. Across 16 studies, exposure to PS particles ranging from 20 nm to approximately 50 µm elicited a spectrum of hepatic responses. Oxidative stress was the most frequently reported outcome (59%), followed by inflammation (41%), apoptosis, mitochondrial dysfunction, lipid metabolism disruption, ferroptosis, and cytotoxicity. Both size- and dose-dependent toxic effects were consistently observed, with NPs demonstrating stronger pro-oxidant and apoptotic activity, while larger MPs often exerted mechanical and lipotoxic effects. Although exposure durations varied (1–72 h), most studies reported significant hepatocellular injury even at concentrations approximating environmentally relevant levels.

**TABLE 3:** Summary of experimental evidence on microplastic-induced hepatotoxicity in human liver cell lines.

	Author (year)	Polymer and size	Dose	Effective dose	Duration (h)	Model	Main hepatic indicators	Key findings
1	Zhang et al. (2025b)	PS MPs (100nm, 1, 5 μm)	10, 50, 100, 500 μg/mL	10, 50, 100, 500 μg/mL	24	HepG2 spheres	Oxidative stress	-Altered physiological activity -Increased lactate dehydrogenase (LDH) release -Higher concentrations and larger particle sizes exert greater toxicity on the spheres
2	Xu et al. (2025)	PS NPs (100 μm)	0, 40, 80 and 160 μg/mL	40, 80, 160 μg/mL	24	HepG2	Oxidative damage, Inflammation	-Stimulates inflammatory cytokine secretion -Promotes lipid synthesis
3	Shi et al. (2025)	PS MPs (5 μm)	0.025, 0.25, 2.5, 25, 250 μg/mL	25, 250 μg/mL	48	Human hepatocyte cells	Oxidative stress	-Activated ferroptosis to regulate cell senescence -Inducing cell senescence
4	Li et al. (2025a)	PS NPs (500 μm)	500 μg/mL	500 μg/mL	24, 48	HepG2	NAFLD and inflammation	-Regulate cell death-associated genes BAX and CASP8
5	Ahn et al. (2025)	PS NPs (50 nm)	0, 10, 20, 50, 100 μg/mL	0, 10, 20, 50, 100 μg/mL	24 to 48	HepaRG	Oxidative stress	-Increase acidic vesicles -Stimulate lipid accumulation (MASLD development)
6	He et al. (2024)	PS MPs (20, 50, 200, 500 nm)	10, 100 mg/mL	10, 100 mg/mL	24	HepG2	Apoptosis	-Induced hepatotoxicity -Size-dependent toxicity of PS at the same concentration, the smaller the size of PS, the greater the enhancement of the apoptotic effect caused by it at the same concentration
7	Guraka et al. (2024)	PS MPs (1, 5 μm)	High dose 25–200 μg/mL Low dose 3.125–25 μg/mL	12.5 and 25 μg/mL	48	hLiMTs	Cell death Inflammation	-Long-term accumulation and adverse effects of non-biodegradable plastics within the liver
8	Guo et al. (2024)	PS NPs (20 nm)	0, 6.25, 12.5, 25 and 50 μg/mL	12.5, 25 and 50 μg/mL	12, 24	HepG2 and L02* cells	Inflammation Oxidative stress	-Decreased survival rates -Induced inflammatory responses -Induced ROS-Nrf2-NF-κB which damage HepG2 cells
9	Fan et al. (2024)	PS NPs (50, 100, 200 nm)	0, 3.125, 6.25, 12.5, 25, and 50 μg/mL	6.25, 12.5, 25, 50 μg/mL	24	L-02*	Oxidative stress Lipophagy	-Induce the accumulation of lipid droplets, with autophagy inhibition exacerbating -Activate lipophagy through the AMPK/ULK1 pathway -Decreased cell viability
10	Chen et al. (2024a)	PS MPs (0.1, 1 μm)	1, 2, 5, 10, 20, 50, and 100 μg/mL	50, 100 μg/mL	48	THLE-2	Cytotoxicity	-Induces chronic toxicity at environmentally relevant concentrations
11	Boran et al. (2024)	PS MPs (3–10 μm)	0.25, 0.5, 1 mg/mL	0.5, 1 mg/mL	72	HepG2/THP-1 co-culture model	Chronic inflammation, Oxidative stress Cytotoxic cell damage	-Causes increased ROS, MDA levels, and protein oxidation -Decreased antioxidant capacity (GSH depletion and reduced SOD2 activity), likely due to mechanical damage from the particles, leading to hepatotoxicity
12	Li et al. (2023a)	PS NPs (20, 60, 100, 500, 1,000 nm)	1, 5, 25, 75, and 125 μg/mL	5, 25, 75, 125 μg/mL	24	LO2 cells*	Oxidative stress	-Oxidative stress induces mitochondrial and cell membrane damage -Reduced ATP production -Increased membrane permeability
13	Li et al. (2023b)	PS NPs (20 nm)	6.25, 12.5, 25 and 50 μg/mL	12.5, 25, 50 μg/mL	24	HepG2	Oxidative stress Cytotoxicity Apoptosis	-Stimulated excessive cellular ROS production -Induced mitochondrial fission by upregulating DRP1 and P-DRP1, while downregulating OPA1 and PGC-1α expression
14	Shen et al. (2022)	PS NPs (100 nm)	1000 μg/L	1000 μg/L	24	HL7702 cells*	Cytotoxicity Inflammation	-Induces nuclear damage and micronucleus formation in hepatocytes, leading to fibrosis
15	Menéndez-Pedriza et al. (2022)	PS MPs (40–48 µm)	49 to 25, 000 mg/L	49 to 25,000 μg/L	48	HepG2	Lipophilic toxic effect	-Induced significant lipidomic changes, particularly in glycerophospholipids and glycerolipids
16	Lin et al. (2022)	PS NPs (80 nm)	6,000, 12,500, 31,250, 62,500, 125,000, 250,000 μg/L	125,000, or 250,000 μg/L	48	L02* cells	Oxidative stress	-Mitochondrial damage -Disruption of metabolic toxicity pathways
17	Banerjee et al. (2022)	PS MPs, NPs (50, 100 nm)	0.1–100 μg/mL	0.1–100 μg/mL	1–24	HepG2	Cytotoxicity Inflammation Apoptosis	-The smaller aminated particles were most toxic to hepatocytes -The larger particles induced apoptosis or an inflammatory response depending on exposure duration

MPs, microplastics (>1 μm) and NPs, nanoplastics (<1 μm); LO, liver organoid; NPs, nanoplastics; MPs, Microplastics; PS, polystyrene; PP, polypropylene; hiPSCs, human-induced pluripotent stem cells; hLiMTs, 3D human liver microtissue; ROS, reactive oxygen species. * LO2 cells, also known as L02, L-02, or HL-7702, were originally believed to originate from a normal fetal liver. However, they are actually a HeLa derivative (Leonard S et al., 2024; Nihart et al., 2025).

Li et al. (2025a) reported that exposure to MPs predominantly modulates cell death–associated genes involved in NAFLD and inflammation, potentially contributing to progression from NAFLD to liver cancer, especially when combined with co-exposures such as cadmium. In addition, exposure to PS-NPs may impair lysosomal degradation, exacerbating hepatocellular injury. This effect is particularly pronounced in fatty liver cells, where PS-NPs promote endocytic lipid accumulation and worsen lipid dysregulation under high-fat diet conditions (Ahn et al., 2025).

Among the studies included, HepG2 cells were the most frequently utilized hepatic model, accounting for approximately 45% of the studies, and they yielded the most consistent findings regarding the mechanisms of MP-induced hepatotoxicity. In multiple studies (Zhang H. et al., 2025; Xu et al., 2025; Li et al., 2025a; He et al., 2024; Guo et al., 2024; Boran et al., 2024; Li et al., 2023b; Menéndez-Pedriza et al., 2022; Banerjee et al., 2022), exposure of HepG2 cells to both polystyrene micro- and nanoplastics consistently resulted in oxidative stress, mitochondrial dysfunction, and apoptosis. This led to downstream activation of pathways such as ROS-Nrf2-NF-κB and DRP1-mediated mitochondrial fission. These reproducible outcomes suggest that oxidative stress-related mechanisms represent a core cytotoxic response in this model.

In contrast, L-02/LO2 cells (used in approximately 20% of the studies) also demonstrated oxidative stress and metabolic impairment, including lipid accumulation and autophagy dysregulation. However, there was a greater focus on lipophagy and AMPK/ULK1 pathway perturbations in these cells (Fan et al., 2024; Li et al., 2023a; Lin et al., 2022). HepaRG and THLE-2 models, which more closely mimic differentiated hepatocytes, revealed similar oxidative and inflammatory responses but were used less frequently, limiting direct comparison (Ahn et al., 2025; Chen Z. et al., 2024).

#### 3.3.2 Experimental evidence of microplastic toxicity in human liver organoids

Recent experimental evidence demonstrates that exposure to PS-MPs, including aged forms, induces pronounced hepatotoxic effects in human liver organoid models derived from pluripotent stem cells (hiPSCs) or embryonic stem cells (Table 4). Six studies investigated PS-MPs ranging from 1 to 10 μm at environmentally relevant and supra-environmental concentrations, with exposure durations ranging from 24 to 500 h (Cheng et al., 2025; Liang et al., 2024; Cheng et al., 2024a; Cheng et al., 2024b; Cheng et al., 2023; Cheng et al., 2022). All human liver organoid models analyzed were developed using three-dimensional (3D) culture systems.

**TABLE 4:** General outcomes of experimental investigations into the cellular and molecular effects of microplastics on human liver cell organoids.

	Author (year)	Polymer and size	Dose	Effective dose	Duration (h)	Model	Main hepatic indicators	Key findings
1	Cheng et al. (2025)	PS, aged PS, MPs (7.60, 6.91 μm)	75 ng/mL	75 ng/mL	500	LO by H9 embryonic stem cell-derived hepatocytes	Metabolic disruptions	-Increased serum Cys -Decreased hepatic Cys -Disturbance in the Hcy metabolism in the liver
2	Liang et al. (2024)	PS MPs (1 μm)	600 ng/mL	600 ng/mL	36	bioprinting hiPSC-derived organoids	Hepatotoxicity	-Changes in liver functions
3	Cheng et al. (2024a)	PS, aged PS MPs (1 μm)	20, 50, 100, 200 μg/mL	50, 100, 200 μg/mL	24	LO by hiPSCs	Hepatocytoxicity	-Aged PS was more cytotoxic than pristine PS -Disrupted iron homeostasis -Abnormal mitochondrial morphology -Elevated lipid peroxidation -Declined GSH peroxidase activity
4	Cheng et al. (2024b)	aged PS MPs (1–10 μm)	5.94–13.15 ng/mL	5.94–13.15 ng/mL	24	LO by hiPSCs	Hepatic reductive stress Hepatoxicity	-Disrupted the genes encoding nutrient transporters and NADH subunits -Restricted ATP production -Decreased mitochondrial membrane potential -Impaired complex I/IV activities -Increased lactate and triglyceride levels
5	Cheng et al. (2023)	PS MPs (1 μm)	10, 100, 500, 1,000, 5,000, 10,000, 50,000 ng/mL	500, 1,000 ng/mL	72	LO by hiPSCs	Hepatotoxicity Lipid accumulation	-Interfered with gene panels involved in multiple lipid metabolism processes - Interfered the proteins production
6	Cheng et al. (2022)	PS MPs (1 μm)	0.25, 2.5 and 25 μg/mL	0.25, 2.5 and 25 μg/mL	48	LO by hiPSCs	Hepatotoxicity Lipotoxicity Cytotoxicity Oxidative stress Inflammation	-Altered levels of key molecular markers - Altered ATP production - Altered lipid metabolism -Induced ROS generation

LO, liver organoid; NPs, nanoplastics; MPs, Microplastics; PS, polystyrene; hiPSCs, human-induced pluripotent stem cells; hLiMTs, 3D human liver microtissues; ROS, reactive oxygen species.

Cheng et al. (2025) reported that prolonged exposure (500 h) to 7–8 μm PS and aged PS MPs at 75 ng/mL disrupted sulfur amino acid metabolism, leading to increased serum cysteine, decreased hepatic cysteine, and perturbed homocysteine pathways, indicating systemic metabolic disturbances. Similarly, Liang et al. (2024) demonstrated that a 36-h exposure of bioprinted hiPSC-derived liver organoids to 1 μm PS MPs (600 ng/mL) resulted in significant alterations in liver function markers (Liang et al., 2024).

Comparative studies revealed that aged PS particles had higher cytotoxicity than pristine PS. Cheng et al. (2024) observed that exposure to 1 μm aged PS MPs (50–200 μg/mL) disrupted iron homeostasis, induced abnormal mitochondrial morphology, increased lipid peroxidation, and reduced glutathione peroxidase activity, collectively indicating oxidative stress and impaired redox regulation (Cheng et al., 2024a; Cheng et al., 2024b). Additional studies confirmed that exposure to 1–10 μm aged MPs at low nanogram concentrations impaired nutrient transporter and NADH subunit gene expression, restricted ATP production, decreased mitochondrial membrane potential, and compromised complex I/IV activity, leading to elevated lactate and triglyceride accumulation (Cheng et al., 2023; Cheng et al., 2022).

Prolonged exposure of hiPSC-derived liver organoids to 500–1,000 ng/mL PS MPs over 72 h interfered with multiple gene panels regulating lipid metabolism and associated protein expression, resulting in significant hepatocellular lipid accumulation. Moreover, showed that PS-MPs induced lipotoxicity, cytotoxicity, oxidative stress, and inflammation, along with altered ATP production, disrupted lipid metabolism, and increased reactive oxygen species generation (Cheng et al., 2022). These studies provide compelling evidence that PS-MPs, particularly aged particles, disrupt multiple metabolic and mitochondrial pathways in human liver organoids, leading to oxidative stress, lipid dysregulation, and hepatocellular injury. These findings underscore the importance of advanced organoid models in assessing human liver responses to environmental contaminants.

#### 3.3.3 Human evidence on polystyrene microplastic exposure and liver health

Emerging human data suggest that exposure to PS-MPs may have measurable effects on liver health (Table 5). Investigated placental exposure to PS-MPs in a cohort of 1,057 pregnant women and found that maternal exposure was associated with changes in fetal liver enzyme activity, including increased levels of alkaline phosphatase, aspartate aminotransferase, and γ-glutamyl transferase. These findings suggest that the transfer of PS-MPs through the placenta may disrupt fetal hepatic function, potentially impacting liver development and metabolic programming (Wa et al., 2025). Analyzed liver tissue from six patients with cirrhosis and detected PS-MPs at concentrations of 4.6–11.9 particles per Gram of tissue. They identified six distinct MP polymers, ranging from 4 to 30 μm in size (Horvatits et al., 2022). Importantly, cirrhotic livers showed a higher burden of MPs compared to non-diseased livers, indicating that chronic liver injury may promote the accumulation or retention of MPs. These studies provide initial but compelling evidence that PS-MPs can reach the human liver, potentially affecting hepatic enzyme activity and contributing to the pathology of chronic liver disease. While the current data is limited, it highlights the necessity for further large-scale investigations to clarify the mechanistic connections between MP exposure and liver health in humans.

**TABLE 5:** Human evidence on polystyrene microplastic (PS-MP) exposure and liver health outcomes.

	Author (year)	Polymer and size	Exposure route	Model	Main hepatic indicators	Key findings
1	Wa et al. (2025)	PS Placental MPs	oral (pregnancy)	Placental sample (N = 1,057)	Change of hepatic function	-Increasing ALP, AST, GGT in fetal liver
2	Horvatits et al. (2022)	PS MPs	4.6–11.9 particles/g (found in liver samples)	Patients with liver cirrhosis (N = 6)	Cirrhosis	-Six different microplastic polymers, ranging from 4 to 30 µm, were identified -Higher MP burden in cirrhotic vs. healthy livers

Abbreviations used: GGT, gamma-glutamyl transferase; ALP, alkaline phosphatase; AST, aspartate aminotransferase; MP(s), Microplastic(s); PS, polystyrene.

## 4 Discussion

### 4.1 Overview of exposure and general findings

**FIGURE 2:**
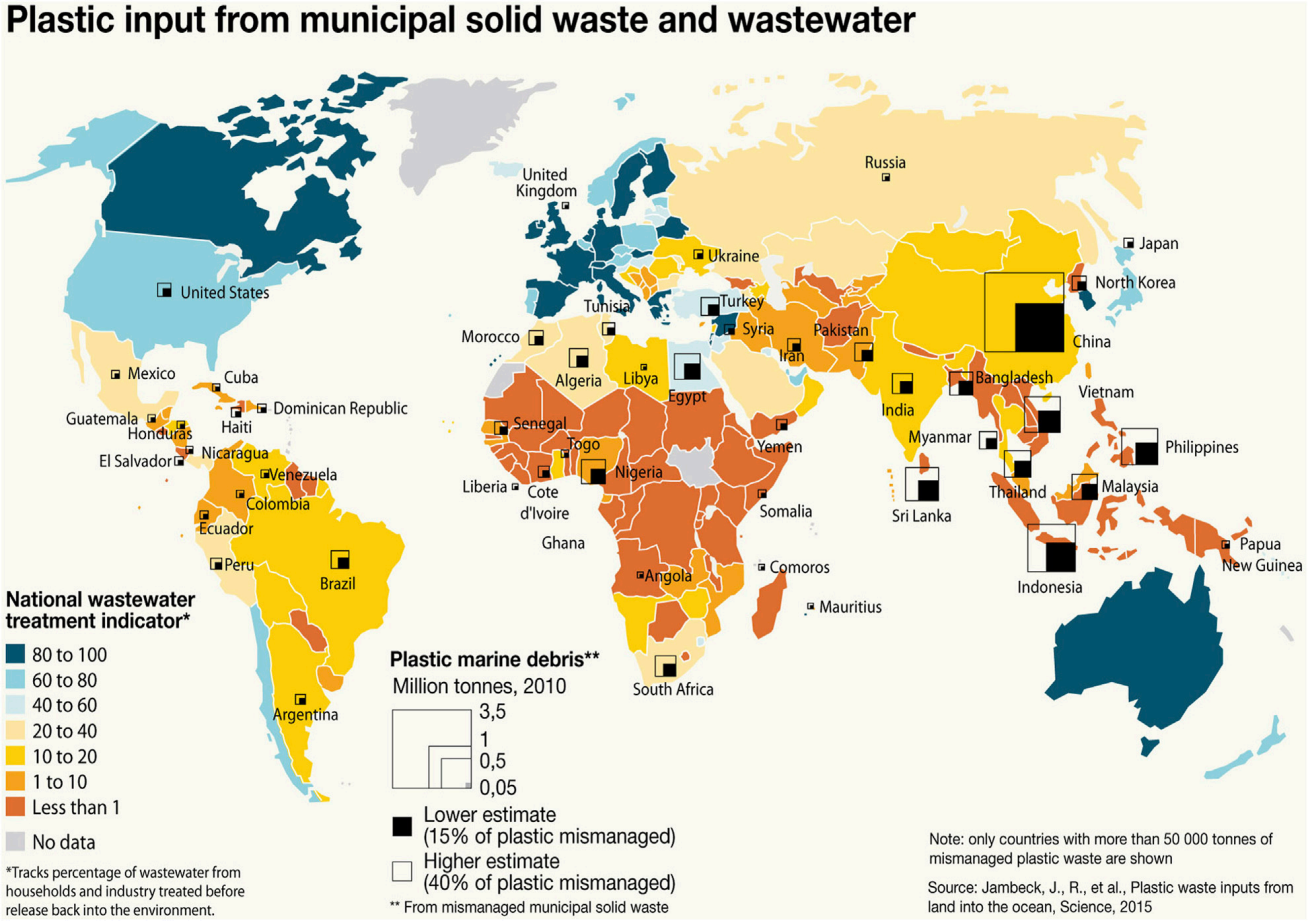
Global plastic inputs originating from municipal solid waste and wastewater across different regions of the world. Data source: GRID-Arendal (https://www.grida.no/resources/6925.

MPs and NPs are now recognized as pervasive contaminants throughout the biosphere, raising growing concerns about their potential effects on human health. Mounting evidence indicates that humans are continually exposed to these particles, primarily through inhalation and ingestion pathways (Ding et al., 2024; Chen J. et al., 2024). A global assessment reported that in 2010, approximately 275 million metric tons of plastic waste were produced across 192 coastal nations, of which an estimated 4.8–12.7 million metric tons entered the marine environment (Jambeck et al., 2015) (Figure 2). The extent of this leakage is largely governed by population density and the efficiency of national waste management systems.

**FIGURE 3:**
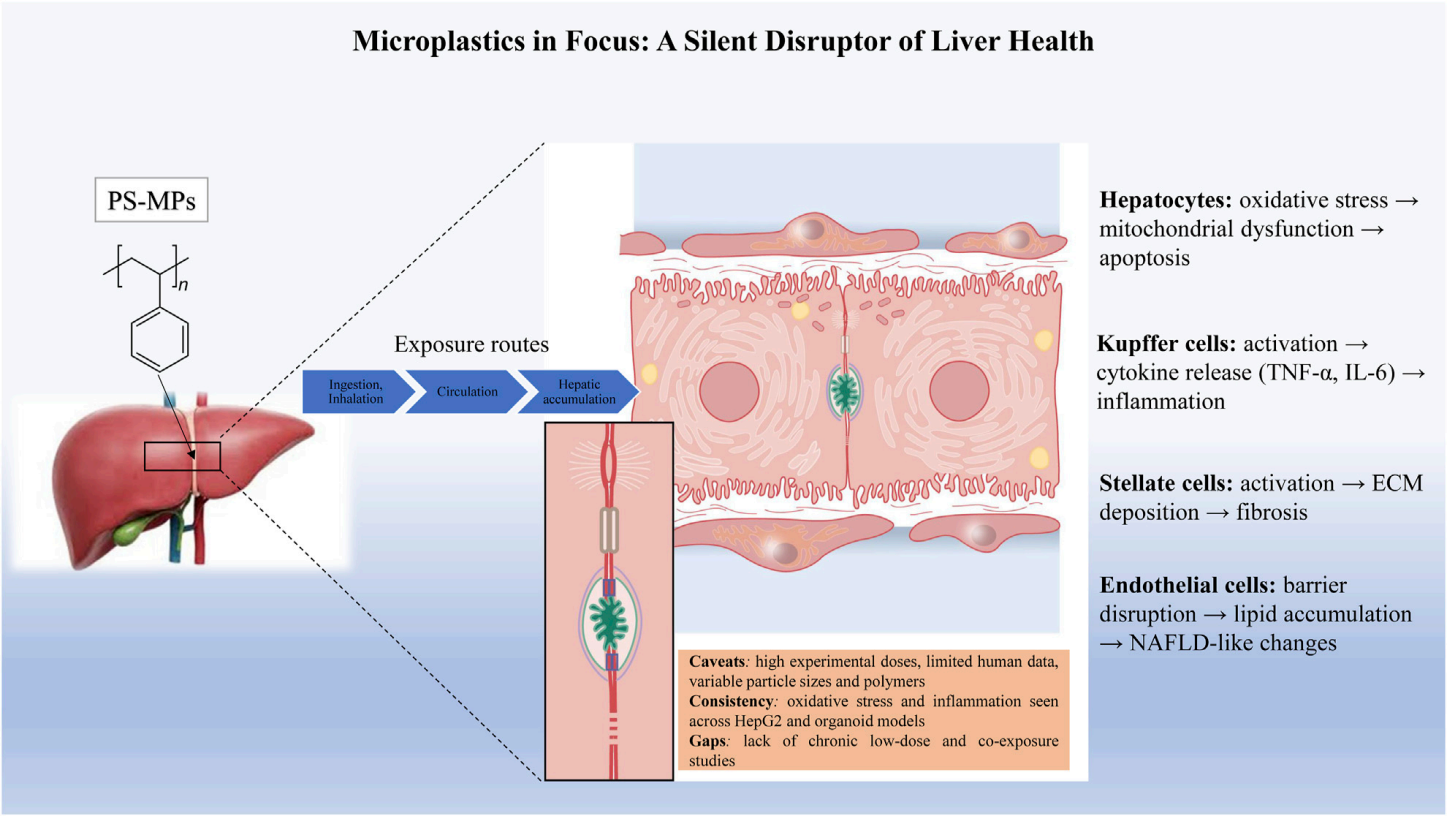
Microplastics as a silent disruptor of liver health. Polystyrene microplastics (PS-MPs) accumulate in the liver, where they can induce oxidative stress, apoptosis, inflammation, and fibrotic/cirrhotic toxicity. The severity of damage depends on the size, concentration, and duration of exposure to microplastics. The inset highlights microplastic interaction at the cellular level.

Our systematic review demonstrated that exposure to MPs exerts significant adverse effects on human hepatic morphology, oxidative balance, cellular integrity, inflammatory responses, and lipid metabolism. The findings further emphasize the influence of key exposure parameters, including life stage, particle size, concentration, and duration of exposure (Figure 3). To contextualize these results, previous experimental studies in animal models have also reported that MP exposure can induce pronounced histopathological alterations in hepatic tissue, such as vacuolar degeneration, chronic inflammatory infiltration, hepatocellular edema, hypertrophy, and hyperplasia (Zheng et al., 2025a; Zheng et al., 2025b; Zheng and Wang, 2025; Zheng PC. et al., 2025; Zhang X. et al., 2025; Zhang J. et al., 2025). While these animal studies were not included in our systematic analysis, they provide valuable *in vivo* evidence that complements *in vitro* and organoid findings, helping to validate the biological relevance of observed effects in human-based models. Together, these data suggest that chronic MP exposure may drive progressive hepatic enlargement and structural degradation over time, and support the use of *in vitro* and organoid systems as predictive models for *in vivo* hepatic responses.

### 4.2 Mechanistic insights into MP-Induced hepatotoxicity

#### 4.2.1 Oxidative stress and mitochondrial dysfunction

Oxidative stress appears to be a central mechanism underlying MP–induced hepatotoxicity, as consistently reported across the majority of cell line, organoid, and human studies included in our review. MPs are capable of interacting with cell surface receptors and penetrating the lipid bilayer, thereby inducing structural perturbations of the cellular membrane (Dai et al., 2022; Fleury and Baulin, 2021). Mitochondria exhibit the greatest degree of dysfunction, displaying morphological alterations, loss of membrane potential, and reduced complex I/IV activities. Beyond the morphological and oxidative damage observed in hepatic tissue and organelles, MP exposure has been shown to impair liver function by disrupting intracellular homeostasis, biotransformation pathways, and lipid metabolism. Common serum biochemical markers, including albumin, alanine aminotransferase, aspartate aminotransferase, alkaline phosphatase, and lactate dehydrogenase, serve as reliable indicators of hepatocellular toxicity and functional impairment, findings that were consistently reported across all organoid-based studies (Cheng et al., 2025; Cheng et al., 2024a; Cheng et al., 2024b; Cheng et al., 2023; Cheng et al., 2022). Organoid-based studies provide particularly compelling mechanistic insights, reporting disruption of homocysteine metabolism, restriction of ATP supply, and structural abnormalities in mitochondrial cristae. These findings suggest that MPs compromise both energy homeostasis and hepatic metabolic integrity, with potential downstream consequences for systemic metabolism (Lin et al., 2022; Cheng et al., 2024a; Cheng et al., 2024b).

Therefore, these findings suggest that oxidative stress is a primary factor in MP-induced hepatocellular damage. It is important to note, however, that most experimental studies used MP concentrations many times higher than those found in human tissues (Leonard S et al., 2024; Nihart et al., 2025). This raises questions about the actual relevance of these oxidative and mitochondrial effects to human pathophysiology. While multiple independent studies support mitochondrial damage as a common mechanism, further research using doses that are more physiologically relevant is necessary to confirm its significance in real-world scenarios.

#### 4.2.2 Disruption of lipid metabolism and energy homeostasis

Regarding lipid metabolism, the liver plays a central role in mediating the uptake, storage, and utilization of lipids. (Wang et al., 2022). Several studies have reported that exposure to MPs can lead to lipid accumulation in liver cell lines and in humans, potentially accelerating the progression of non-alcoholic fatty liver disease (NAFLD) or metabolic-associated steatotic liver disease (MASLD) (Shi et al., 2025; Ahn et al., 2025; Fan et al., 2024; Menéndez-Pedriza et al., 2022; Cheng et al., 2023). High-dose and prolonged exposures elicit frank cytotoxicity, including apoptosis, necrosis, and micronucleus formation in hepatocytes. Three-dimensional human liver microtissues and induced pluripotent stem cell-derived organoids confirm cumulative and non-linear toxic effects, underscoring the risk of bioaccumulation and persistence of non-biodegradable plastics. Importantly, translational evidence is beginning to emerge: a histological study of human liver samples demonstrated significantly elevated MP concentrations in patients with cirrhosis compared to controls, marking a critical step in linking experimental findings with human pathology (Horvatits et al., 2022). However, the clear mechanisms by which MPs influence hepatic lipid metabolism and cytotoxicity remain largely unclear and warrant further investigation.

The majority of studies support lipid accumulation as a downstream effect of oxidative and mitochondrial disturbances rather than a standalone mechanism. Nevertheless, there is some variation among reports, with different research groups suggesting involvement of PPARα signaling, β-oxidation inhibition, or ER stress as potential mediators. These inconsistencies highlight the need for standardized lipidomics approaches to determine whether MP-induced lipid dysregulation represents a conserved or context-dependent phenomenon.

#### 4.2.3 Inflammatory and apoptotic responses

In addition to oxidative and metabolic alterations, several studies have demonstrated that MP exposure provokes inflammatory and apoptotic responses (Xu et al., 2025; Li et al., 2025a; Guraka et al., 2024; Guo et al., 2024; Boran et al., 2024; Shen et al., 2022; Banerjee et al., 2022). Elevated levels of pro-inflammatory cytokines (e.g., TNF-α, IL-6) and caspase activation have been reported in cell-based and organoid models, indicating secondary pathways of hepatocellular injury (Cheng et al., 2022). These mechanisms appear consistent across multiple studies, but human evidence remains limited. It is plausible that inflammation and apoptosis represent downstream consequences of mitochondrial dysfunction and lipid peroxidation, linking cellular damage to tissue remodeling and fibrosis.

#### 4.2.4 Influence of particle properties on mechanistic outcomes

**FIGURE 4:**
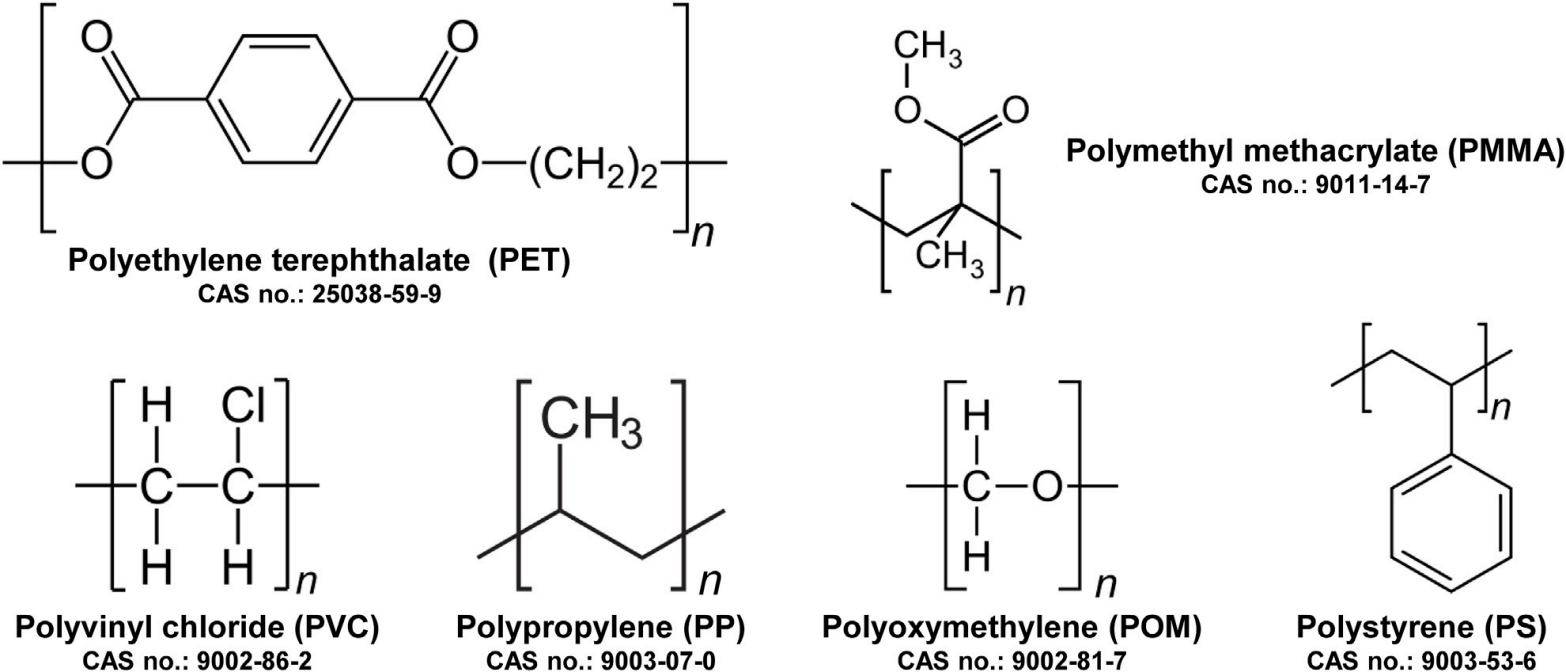
Representative chemical structures of major microplastic polymers frequently detected in environmental and toxicological studies. Polymers included are polyethylene terephthalate (PET), polymethyl methacrylate (PMMA), polyvinyl chloride (PVC), polypropylene (PP), polyoxymethylene (POM), and polystyrene (PS).

The biological impact of MPs is strongly influenced by their physicochemical characteristics, including type, size, shape, aspect ratio, porosity, and surface charge, as well as the exposure concentration (Ebrahimi et al., 2022). Multiple polymer types, including polyethylene terephthalate (PET), polymethyl methacrylate (PMMA), polyvinyl chloride (PVC), polypropylene (PP), polyoxymethylene (POM), and polystyrene (PS), are prevalent MP contaminants in environmental matrices (Figure 4). In this review, we focused specifically on PS particles to maintain methodological consistency and reduce variability arising from differences in polymer composition, surface chemistry, and physicochemical behavior. Notably, PS is among the most widely produced and utilized plastics globally and is frequently detected in both aquatic and terrestrial ecosystems. Its persistence and potential for bioaccumulation make PS a representative model for investigating MP-induced toxicity in biological systems (Jolaosho et al., 2025). Moreover, particle size plays a critical role in determining accumulation patterns, tissue distribution, and ensuing toxicological effects. For example, two studies reported that smaller PS particles elicited more pronounced apoptotic responses at equivalent concentrations, indicating size-dependent toxicity and enhanced hepatotoxicity in the human HepG2 cell line (He et al., 2024; Banerjee et al., 2022). Smaller MPs, owing to their higher surface-area-to-volume ratio, are more readily ingested, accumulate in the gastrointestinal tract, and can translocate to the liver, where they exert increased hepatotoxic effects (Jeong et al., 2016). This phenomenon is partially attributable to differences in surface charge, which influence particle uptake and intracellular trafficking within endosomes (Dausend et al., 2008). Furthermore, aged PS demonstrated higher cytotoxic potential compared with newly manufactured PS in organoid models (Cheng et al., 2025; Cheng et al., 2024a; Cheng et al., 2024b). Moreover, MPs of different polymer types exhibit variations in chemical stability, degradation rates, and pollutant adsorption capacity, which further modulate their ecotoxicological profiles (Li et al., 2025b). Among laboratory studies, PS-MPs are the most commonly utilized model particles and have been shown to exert broad and significant effects on hepatic tissues, reinforcing their utility in mechanistic studies of MP toxicity.

The concentration of MPs is a critical determinant of their biological impact. In this study, we observed associations between MPs exposure levels and liver-related outcomes. The results demonstrated clear dose–response relationships, with higher concentrations of MPs corresponding to increases in multiple hepatic indicators within specific exposure ranges. While *in vitro* studies consistently report dose-dependent hepatotoxic effects, many of these experiments use supraphysiological concentrations that far exceed levels detected in human tissues. For example, some studies used PS-MP doses of 10–100 mg/mL (He et al., 2024), while MPs found in human blood are typically around 5 μg/mL (Leonard S et al., 2024), and liver tissue concentrations are even lower (∼3–10 particles per Gram) (Nihart et al., 2025). This significant difference raises concerns about the direct applicability of *in vitro* findings to human pathophysiology. Thus, the observed cytotoxicity, oxidative stress, and lipid dysregulation in cell lines may be a result of extreme experimental conditions rather than realistic, physiological exposures. To enhance the translational relevance of future research, there is a necessity for standardized experimental designs that utilize environmentally and physiologically relevant concentrations. This would allow for more precise evaluation of MP-induced hepatotoxicity in humans.

## 5 Study limitations and research needs

This systematic review has several notable limitations. First, the available evidence on MPs toxicity in human liver models remains very limited, with only two studies conducted in human liver, which restricts the generalizability of the findings. Second, of the six organoid studies included, five were derived from a single research center and authored by the same group, raising concerns about potential bias and limiting the diversity of experimental approaches and populations. Third, the limited number of studies, coupled with variability in types of MPs, concentrations, and exposure durations, prevent the conduct of meta-analytical synthesis and complicates the derivation of definitive conclusions regarding dose–response relationships or underlying mechanistic pathways. Fourth, the absence of standardized protocols for MPs exposure, reporting metrics, and organoid characterization further reduces comparability across studies. Fifth, most available studies, including the limited human data, originate from Asian regions, which may introduce geographical bias. The lack of broader regional representation and model diversity limits the generalizability of the findings to global populations. Finally, since most included studies were case-control in design, the influence of unmeasured confounding factors cannot be entirely ruled out. Therefore, these limitations underscore the need for multi-center, standardized, and larger-scale investigations to strengthen the evidence base on MPs-induced hepatotoxicity in human-relevant models. It should be noted that some studies included in this review employed the LO2/L-02 cell line, which has been reported to be a HeLa derivative rather than a normal hepatocyte line. Therefore, results derived from these models should be interpreted with caution, as they may not fully represent normal hepatic physiology (Shao and Chen, 2024; Weiskirchen, 2023).

The current human evidence on MP exposure and liver health is limited, with only a few studies available (Wa et al., 2025; Horvatits et al., 2022). One of the few human studies investigating MP accumulation in the liver was a proof-of-concept case series conducted in Germany, which analyzed tissue samples from six patients with liver cirrhosis and five individuals without underlying liver disease (Horvatits et al., 2022). Using a combination of chemical digestion, Nile red staining, fluorescent microscopy, and Raman spectroscopy, six different MP polymers ranging from 4 to 30 µm were detected in cirrhotic livers but not in control samples. While these findings suggest that MPs may accumulate in diseased livers, several key details were not reported, including the etiology of liver cirrhosis for each patient, circulating MP concentrations, and potential differences in particle size among etiologies. These limitations should be considered as caveats, highlighting the need for larger, more detailed studies to clarify whether hepatic MP accumulation contributes to disease pathogenesis or is a consequence of cirrhosis and portal hypertension. Nevertheless, the detection of MPs in human liver tissue provides critical translational support for the mechanisms identified in in vitro and organoid studies, particularly oxidative stress, mitochondrial injury, and lipid dysregulation. The convergence of these experimental and clinical findings strengthens the hypothesis that chronic low-level MP exposure may contribute to subtle hepatic dysfunction in humans.

To better understand potential health effects, there is a clear need for well-designed epidemiological studies that assess exposure levels, account for confounding factors, and examine long-term hepatic outcomes across diverse populations. The human studies included in this review were conducted under ethical guidelines to ensure participant safety and informed consent. However, ethical constraints may limit experimental interventions, sample collection, or exposure assessments, potentially reducing mechanistic insights. Moreover, co-exposures to other environmental chemicals, lifestyle factors, or dietary components may act as confounders, influencing observed hepatic outcomes and complicating the interpretation of MP-specific effects. Hence, current human data remain largely descriptive and require integration with mechanistic evidence from controlled experimental systems to establish causal links between exposure and hepatocellular injury. These considerations should be taken into account when extrapolating findings and planning future research.

Across the 24 *in vitro* studies included in this review, PS-MPs and PS-NPs were tested at a wide range of concentrations, from as low as 0.025 μg/mL to as high as 25,000 μg/mL, with exposure durations ranging from 1 to 72 h. The most commonly used human hepatic cell lines were HepG2, LO2/L-02, HepaRG, THLE-2, and co-culture models. In the human study, MP concentrations in liver tissue were relatively low, with median values of 4.6 particles per Gram tissue (3.2 particles per Gram after blank correction) in patients with cirrhosis, and 0.0–1.5 particles per Gram in patients without liver disease. In contrast, *in vitro* studies included in this review exposed human hepatic cells to much higher concentrations, ranging from 0.025 μg/mL to over 25,000 μg/mL. This indicates that most *in vitro* experiments used supraphysiological doses, likely designed to elicit measurable cellular responses such as oxidative stress, apoptosis, inflammation, and lipid dysregulation. While these studies are valuable for elucidating mechanisms of hepatotoxicity, the substantial discrepancy between experimental and physiological concentrations underscores the need for future *in vitro* studies that more closely mimic the low-level exposure observed in human tissues, to improve relevance to human pathophysiology. This comparison also highlights that while multiple studies corroborate the oxidative and metabolic pathways of toxicity, their quantitative contribution under realistic human exposure conditions remains to be established.

## 6 Conclusion

In conclusion, emerging evidence underscores that PS-MPs and PS-NPs compromise liver function by disrupting redox equilibrium, impairing metabolic processes, and altering tissue architecture in cellular, organoid, and limited human models. The current literature, however, is constrained by variability in particle characterization, exposure conditions, and outcome measures, as well as a scarcity of human data, which collectively limit precise risk quantification. Addressing these gaps will require harmonized experimental frameworks, longitudinal human studies, and mechanistic investigations to clarify dose–response relationships, identify vulnerable populations, and guide strategies to mitigate MP-induced hepatotoxicity.

## Data Availability

The original contributions presented in the study are included in the article/Supplementary Material, further inquiries can be directed to the corresponding authors.

## Abbreviations

hiPSC, Human induced pluripotent stem cells; LDH, Lactate dehydrogenase; LD(s), Lipid droplet(s); MP(s), Microplastic(s); NAFLD, Non-alcoholic fatty liver disease; NPs, Nanoplastics; PET, Polyethylene terephthalate; PMMA, Polymethyl methacrylate; PP, Polypropylene; POM, Polyoxymethylene; PS, Polystyrene; PS-MPs, Polystyrene microplastics; PS-MPs/NPs, Polystyrene micro- and nanoplastics; PS-NPs, Polystyrene nanoplastics; TG, Triglyceride; PVC, Polyvinyl chloride.
